# Clinical Advances in Kidney Failure: AKI

**DOI:** 10.3390/jcm12051873

**Published:** 2023-02-27

**Authors:** Alaa S. Awad, Emaad M. Abdel-Rahman

**Affiliations:** 1 Division of Nephrology, University of Florida, Jacksonville, FL 32209, USA; 2 Division of Nephrology, University of Virginia, Charlottesville, VA 22908, USA

Kidney failure poses an enormous burden on patients, caregivers, healthcare providers, and society as a whole. While the etiology and management of kidney failure differ between low- and high-income countries, epidemiological studies point to the continued increase in the numbers of patients with kidney failure worldwide. Two types of kidney failure exist: acute kidney injury (AKI) and chronic kidney disease (CKD). The current Special Issue of the *Journal of Clinical Medicine*, “Clinical Advances in Kidney Failure”, is mainly focused on AKI.

Despite the vast range of literature focusing on AKI, several gaps still exist pertaining to the optimum care of AKI patients. The diverse etiologies, mechanisms, settings, and outcomes of AKI pose a challenge to the unified, standardized, and optimum management of these patients. A step-by-step review of AKI, from prevention of the disease to its etiology, pathogenesis, diagnosis, and management, needs to be routinely updated to identify opportunities and hopefully provide suggestions to bridge these gaps.

Novel therapeutic interventions for preventing or attenuating renal tissue injury following AKI remain a focus of significant interest. AKI is a serious complication affecting >5–7% of hospitalized patients and is associated with a high mortality, morbidity, and increased healthcare costs [1,2,3,4]. Furthermore, 26% of patients with AKI superimposed on CKD die during hospitalization, and between 42 and 63% develop end-stage kidney disease (ESKD) [5].

The etiology and pathogenesis of AKI involve multifactorial processes including, but not limited to, renal ischemia, nephrotoxicity, volume depletion, and obstructive uropathy. In the current issue, Turget et al. highlight the different causes of AKI along with the mechanism(s) of renal injury [6]. Several cascades initiated by AKI causing tissue injury, such as changes in renal blood flow, hypoxic cell death, adenosine triphosphate (ATP) depletion, vascular endothelial cell injury, mitochondrial dysfunction, tubular epithelial cell injury, and leukocyte recruitment, result in altered kidney homeostasis [7,8,9,10,11]. Currently, there are no definitive therapeutic or protective approaches available for AKI. Thus, it is important to identify the mechanisms involved in the development and progression of AKI.

Diagnostic criteria of AKI have been developed for the identification and monitoring of AKI. The three most commonly used criteria are KDIGO, RIFLE, and AKIN criteria [12]. However, the diagnosis of AKI mainly relies on assessing the serum creatinine and urine output. Neither are sensitive nor timely in reporting the state of kidney damage. Kinetic eGFR, as presented in the current issue [13], could provide a sensitive tool in the assessment and the severity of kidney function following AKI, especially in acute-pancreatitis-induced AKI.

Racial disparities in the clinical outcomes of several health conditions are very common, especially in AKI patients, as Black patients have a higher risk of AKI compared to non-Black patients. Several factors account for these differences, including susceptibility to kidney injury, severity of illness, and socioeconomic factors. In this current Special Issue [14], the authors further explore the link between race and AKI using the incidence, diagnosis, and management of AKI to illustrate how race is directly related to AKI outcomes, with a focus on Black and White individuals with AKI.

Several therapeutic targets have been proposed to offset AKI morbidity and mortality. For instance, early treatment using enzyme replacement therapy with agalsidase alfa induces renal tissue protection by reducing proteinuria in patients with Fabry disease [15].

While early recovery of AKI can occur before discharge, mortality remains very high, especially in patients requiring dialysis during hospitalization. After discharge, AKI patients could have complete resolution of kidney injury and be diagnosed with CKD, requiring dialysis AKI, or declared ESKD. Therefore, optimum care for AKI survivors requires a multidisciplinary approach and ideally should start while patients are hospitalized. Patients should continue as outpatients, as described in detail in the current Special Issue [16]. Care should be individualized depending on the patient’s demographics, risk of recurrence, and severity of AKI. Spectra of care could include patient-centered outcomes, potential interventions, and intermediate and long-term clinical outcomes.

Identifying modifiable predictors of outcomes for AKI patients requiring hemodialysis (AKI-D) will improve care and outcomes, as presented in the current Special Issue [17]. Specifically, optimizing dialysis prescription to decrease the frequency of hypotension during dialysis, minimizing excessive ultrafiltration, and close monitoring of outpatient AKI dialysis are crucial and may improve outcomes for these patients.

Whether peritoneal dialysis is an equivalent option to hemodialysis for AKI patients is not completely clear, mainly due to its limited use and limited clinical trials. In this Special Issue, Dr. S. Khan [18] highlights the current data available on the utilization of peritoneal dialysis following AKI.

In summary, the aim of this Special Issue is to highlight the recent advances pertaining to earlier diagnosis, predicting outcomes, and better management of AKI in the hospital setting and post-hospital discharge.

As the Guest Editors, we sincerely appreciate and thank the reviewers for their insightful remarks and the *JCM* team’s support. We also heartily thank all of the contributing authors for their valuable contributions.
